# Comment on Nahok et al. Monosodium Glutamate Induces Changes in Hepatic and Renal Metabolic Profiles and Gut Microbiome of Wistar Rats. *Nutrients* 2021, *13*, 1865

**DOI:** 10.3390/nu14204386

**Published:** 2022-10-19

**Authors:** Huichia Chao, Shintaro Yoshida, Masanori Kohmura

**Affiliations:** International Glutamate Technical Committee (IGTC), Washington, DC 20045, USA

This letter is to comment on the study of Nahok, K. et al. [1], who report that MSG consumption induces hepatic and renal metabolic changes together with gut microbiota compositional shifts, which may be associated with adverse effects with the long-term use of MSG. 

The authors cited Pen, Q. et al.’s study, in which it was concluded that the effect of MSG on human microbiota was limited because the impact of individuals was greater than that of MSG [2]. However, the authors speculated that “gut microbiota in humans was not significantly changed due to the low dose of MSG supplementation (2 g/day), since the average daily MSG intake in Thailand is 4 g/day [3]”. This is obviously being over-speculative, because Insawang, T. et al. did not provide any data on the association between MSG intake and changes in microbiota [3]. In addition, the MSG intake in this study can be calculated as 1.5 g/kg bw/day, corresponding to an unrealistic dose in humans of 90 g/60 kg bw/day. Dietary glutamate is also derived from foods as protein-bound and free glutamate, and Schmit, J. A. et al. reported that the average background intake of glutamate is estimated at around 15 g/day [4], which is far less than 90 g/day. Taking the total glutamate in foods into consideration, even if the daily intake of MSG supplementation increases, its impact should be considered too small to alter the human microbiota.

It is established that glutamate ingested with food is metabolized as an energy source for enterocytes. The metabolic fate in rat small intestine showed that most glutamate is catabolized to CO_2_ (64%), lactate (16%), proline (4.1%), etc. [5,6], meaning most dietary glutamate is utilized by the small intestine rather than passing to the liver. In this report, it is also shown that there were no significant differences in the metabolites found in the jejunum, feces and plasma in the MSG treatment groups, explaining that the amount of dietary glutamate which can enter the colon is limited. Moreover, trimethylamine (TMA) is mainly formed from phosphatidylcholine/choline and carnitine by microflora in the colon [7,8]; therefore, dietary glutamate is not involved in the biosynthesis of TMA.

Finally, the authors suggest that the metabolite changes in liver and kidney may be associated with adverse effects from the long-term use of MSG. However, it has been demonstrated that high doses of dietary glutamate do not affect liver metabolites [9] and long-term toxicological data at doses of up to 4% in the diet for up to 2 years show no adverse effects of MSG/glutamate on any organs [10]. We argue that dietary MSG does not induce compositional changes in gut microbiota nor the following metabolite changes in the liver or kidney, and the long-term use of MSG does not have any safety concerns.
